# Logistic Regression and Machine Learning Models for Predicting Whether Intensive Care Patients Who Are Alert and Without Delirium Remain As Such for at Least Two More Days

**DOI:** 10.7759/cureus.34913

**Published:** 2023-02-13

**Authors:** Rachel A Hadler, Franklin Dexter, Richard H Epstein

**Affiliations:** 1 Anesthesia, University of Iowa, Iowa City, USA; 2 Anesthesiology, University of Miami Miller School of Medicine, Miami, USA

**Keywords:** psychological distress, managerial epidemiology, patient dignity inventory, palliative medicine, unsupervised machine learning

## Abstract

Background

Some intensive care unit patients are alert and without delirium for at least two consecutive days. These patients, like other critically ill individuals, are at risk for dignity-related distress. An interval of at least two days would provide for a palliative care multidisciplinary team to be consulted in the late morning or afternoon of day one and visit the next day. An assessment would include the administration of the validated Patient Dignity Inventory in a reflective manner. To determine whether dignity-related distress can be identified and treated during patients’ intensive care unit stay, we evaluated whether a substantive fraction of such patients (≥5%) have a substantial (>90%) probability of remaining alert and without delirium in the intensive care unit for at least four consecutive days.

Methods

The retrospective cohort study used data from one large teaching hospital in the United States of America, from 2012 to June 2022. The inclusion criteria were: a) adults, b) present in an intensive care unit at 12 PM one day and continually so for the next 48 hours, c) during those two days had every Riker sedation-agitation scale score “4, calm and cooperative,” and d) during those two days had all Confusion Assessment Method for the Intensive Care Unit (CAM-ICU) scores negative (i.e., no delirium) and all Delirium Observation Screening Scale (DOS) scores less than three (i.e., no delirium).

Results

Among the 10,314 patients alert and without delirium in an intensive care unit over two-day periods that included three successive 12 PMs, 3,826 (37%) maintained this status for at least two successive 12 PMs. Six patient characteristics (e.g., hemodynamic infusion or ventilatory support) had value in predicting those 37% of patients. However, logistic regression and classification models each predicted a few (≈0.2%) patients with >90% probability of maintaining these criteria. Forecasts were inaccurate for nearly all patients remaining alert and without delirium in the intensive care unit (≈37%) because the models predicted no patient alert, without delirium, and in the intensive care unit for two days would remain so for at least four days. That ≈63% accuracy was improved upon by random forest machine learning, but only with ≈3% improvement.

Conclusion

Although many intensive care unit patients remain alert and without delirium for several consecutive days, each patient has a high daily probability of intensive care unit discharge or deterioration in medical condition. Therefore, the results of our prediction modeling show that care models for the assessment and treatment of patients with intensive care unit-associated dignity-related distress should not rely solely on the intensive care unit team but instead should be taken from the perspective of the entire hospitalization.

## Introduction

Many (e.g., 20%) intensive care unit patients have one or more intervals of at least two days in the intensive care unit during which they are alert and without manifestations of delirium [1,2]. This criterion includes patients with severe illness (e.g., viral myocarditis and cardiogenic shock or spinal shock from cervical trauma, both with hemodynamic infusions). The criterion excludes patients admitted overnight for neurological or cardiac monitoring. Despite an outward appearance of being cooperative and oriented, many of these patients have severe emotional, psychological, and existential distress [1]. Dignity-related distress can be so severe that patients desire death over their current condition [3]. Among intensive care unit patients, no combination of demographic factors or treatment types accurately predicts the occurrence of dignity-related distress, suggesting that each intensive care unit patient who is alert and without delirium for two or more days should be assessed for such distress [4]. We are interested in intervals of at least two days, expecting that a palliative care multidisciplinary team would be consulted in the late morning or afternoon of day one and visit the following day to assess the patient. Our interest was to figure out if it is possible to predict accurately patients who have been alert and non-delirious in the intensive care unit for at least two days and will remain so for another two days, reliably providing time for assessment and treatment in the unit.

The University of Manitoba’s 25-item Patient Dignity Inventory includes psychological, physical, and social themes, each scored on a 5-point Likert scale from “not a problem” to “an overwhelming problem” [1,5,6]. The individual items with high scores are heterogeneous among critically ill patients [1], suggesting that no one strategy would uniformly reduce patients’ distress. For example, focusing efforts to reduce the time to answer the call button may result in patients “feel[ing more] supported by [their] health care providers,” but not all patients. Spiritual care consult would benefit patients with “concern that [their] spiritual life is not meaningful,” but would be less effective for patients with other concerns such as functionality. The Patient Dignity Inventory can be administered in a reflective manner (e.g., asking follow-up questions to understand causes of distress and potentially what may be addressed) [7]. Based on the individual patient’s responses, interventions such as counseling, occupational therapy, and/or changes to the environment can then be applied.

Moving from an intensive care unit to a ward may change dignity-related distress (e.g., the “feeling that my illness and care needs have reduced my privacy” [5]). Ideally, treatment of dignity-related distress would start in the intensive care unit, but that requires time to identify the distress and to deploy intervention. Our goal was to learn whether the joint effect of predictors was sufficient to detect a substantive fraction of patients (≥5%) each with a substantial (>90%) probability of remaining alert and without delirium and the intensive care unit for two subsequent consecutive days, conditional on their being alert and without delirium in the unit for the two previous days. With such a prediction, the intensive care unit team could reasonably address dignity-related distress among such patients. On the other hand, if few patients reliably remain in the intensive care unit, different care models for assessment and treatment of dignity-related distress may need to be used (e.g., palliative care team consultation). Ours is a paper about prediction and its accuracy.

## Materials and methods

The University of Iowa Institutional Review Board approved this retrospective cohort study #201911151 on July 7, 2022, without requiring patient consent. The study was performed without the use of data that could result in patient identification.

Although the large US teaching hospital studied started using the Epic electronic health record in 2008 (Epic Systems, Verona, Wisconsin), our study’s functional starting date was the first quarter of 2012, when the first patients met all inclusion criteria. The study end date was the second quarter of 2022, the last full quarter before we started data analysis. The inclusion criteria for the study patients were: a) adults defined as patients aged >17 years on the date of hospital admission, b) present in an intensive care unit at 12 PM one day and continually so for the next 48 hours, c) during those two days had every Riker sedation-agitation scale score “4, calm and cooperative,” and d) during those two days had all Confusion Assessment Method for the Intensive Care Unit (CAM-ICU) scores negative (i.e., no delirium) and all Delirium Observation Screening Scale (DOS) scores less than three (i.e., no delirium). Thus, throughout the paper, when we refer to the patients as being “alert and not delirious,” we mean that whenever the patient was instructed to answer questions, they did so without suggestion of delirium. Although there are no data on how often patients were sleeping and awakened to answer questions or assessment was deferred, alertness was assessed several times daily, and delirium assessments were made at least daily. If the Riker scale was missing, or if both the Confusion Assessment Method and Delirium Observation Screening Scale were missing, the criteria were considered not satisfied. Intubation was not considered to be an exclusion criterion if the documentation, as above, was consistent with the patient’s ability to take part fully in the delirium assessment, and the patient was determined not to be delirious. If there were no evaluation for alertness (Riker) and/or delirium (CAM-ICU, DOS) around 12 PM in a patient, and if the preceding and following evaluations both showed alert and without delirium, the patient was considered alert and not delirious between those assessments and considered to have met the inclusion criteria. However, if the next evaluation after 12 PM showed that the patient either was not alert (Riker scale score not four) or was delirious (CAM-ICU or DOSS positive), the status at the preceding 12 PM was considered indeterminate, and the patient was excluded. Because intensive care unit patients’ conditions change temporally, our criteria included only the first two-day period that the criteria were satisfied for each patient (i.e., each patient was studied only once).

Our primary endpoint was binary: whether the patient who had been alert and without delirium in the intensive care unit over the period including three successive 12 PMs remained alert and without delirium for another two days. In other words, all patients studied had documentation that they were alert and without delirium in an intensive care unit for two successive 24-hour periods starting at 12 PM. We selected 12 PM as the beginning of these intervals based on the consideration that a consultation (e.g., to palliative care service) could be requested and completed in the late afternoon on the first day or early in the morning on the second day. The binary dependent variable was assigned a value of one if the patient was alert (when not sleeping) and without delirium for four days starting at 12 PM (i.e., the period included five successive 12 PMs). The dependent variable was assigned a value of zero if the patient was alert and without delirium over the first three successive 12 PM, but the criteria were not satisfied for any evaluations during the next two days. Our independent variables (Table 1) included demographic factors (e.g., patient sex) and attributes related to the patient’s status over the second of the four 24-hour periods (e.g., ventilatory or pressor support during that period). We focused on the second of the two baseline 24-hour periods under the expectation that a member of the multidisciplinary team would visit the patient during this period, assess dignity-related distress [1], and possibly implement intervention(s).

**Table 1:** Characteristics of intensive care unit (ICU) patients alert and without delirium for two consecutive days, including three successive 12 PM ^a^ The second and third columns are reported either as % (number of patients) for binary data or as median (interquartile range) {mean} for continuous data.

Characteristic over 24 hours, from 12:00 PM to 11:59 AM before third successive 12 PM	Alert no delirium in the ICU <4 days (n=6,488)^a^	Alert no delirium in the ICU ≥4 days (n=3,826)^a^	Cohen’s D (descending sequence)
Any hemodynamic infusion	12% (770)	28% (1063)	0.39
Any ventilatory support	15% (975)	29% (1100)	0.35
Nasal high flow therapy used	6% (414)	14% (533)	0.26
Norepinephrine infusion	6% (391)	13% (505)	0.26
Norepinephrine total dose (mcg)	0 (0, 0) {336}	0 (0, 0) {884}	0.26
Epinephrine infusion	1% (93)	5% (186)	0.21
Epinephrine infusion total dose (mcg)	0 (0, 0) {37}	0 (0, 0) {225}	0.21
Milrinone infusion	1% (76)	4% (165)	0.21
Milrinone infusion total dose (mcg)	0 (0, 0) {274}	0 (0, 0) {1023}	0.21
Mechanical ventilation	7% (451)	12% (454)	0.17
Nicardipine infusion	5% (297)	8% (325)	0.17
Nicardipine infusion total dose (mg)	0 (0, 0) {3.22}	0 (0, 0) {6.86}	0.17
Any mechanical cardiac assist device	2% (119)	5% (174)	0.16
Ventricular assist device	1% (64)	2% (97)	0.12
Operating room (surgery) during preceding 7 days	37% (2424)	32% (1217)	0.12
Cardiac intensive care unit	31% (1980)	35% (1353)	0.104
Hospital days	4.2 (3.2, 6.7) {6.0}	4.4 (3.3, 7.7) {6.6}	0.100
COVID-19 positive during hospitalization	3% (201)	5% (184)	0.09
COVID-19 days during hospitalization	0 (0, 0) {1.3}	0 (0, 0) {1.6}	0.09
Intra-aortic balloon pump	1% (38)	1% (56)	0.09
BiPAP, bilevel positive airway pressure	2% (110)	3% (113)	0.09
Operating room (surgery) during hospitalization	41% (2692)	38% (1446)	0.08
Fentanyl	4% (258)	5% (209)	0.07
Fentanyl total dose (mcg)	0 (0, 0) {50}	0 (0, 0) {66}	0.07
Medical intensive care unit	30% (1915)	26% (1010)	0.07
Hydromorphone	4% (254)	3% (120)	0.04
Hydromorphone total dose (mcg)	0 (0, 0) {375}	0 (0, 0) {312}	0.04
African American/ Black, listed as patient’s race	5% (343)	6% (238)	0.04
Extracorporeal membrane oxygenation	0% (19)	1% (21)	0.04
Year in intensive care unit and alert without delirium	2018 (2016, 2020) {2017}	2018 (2016, 2020) {2017}	0.04
Surgical neurological intensive care unit, the cardiac and medical units listed above	40% (2592)	38% (1463)	0.04
Morphine	2% (147)	3% (106)	0.03
Morphine total dose (mcg)	0 (0, 0) {168}	0 (0, 0) {263}	0.03
White, listed as patient’s race	89% (5796)	88% (3382)	0.03
Any narcotic	25% (1635)	26% (1014)	0.03
Hispanic/ Latino listed as patient’s race	3% (163)	3% (109)	0.02
Asian listed as patient’s race	1% (80)	1% (40)	0.02
Patient age in years	63 (52, 72) {61}	62 (52, 72) {61}	0.01
Vasopressin infusion	2% (123)	4% (165)	0.02
Vasopressin infusion total dose (units)	0 (0, 0) {0.49}	0 (0, 0) {2.40}	0.02
Sequence of case, by date	5179 (2528, 7771) {5146}	5130 (2673, 7667) {5177}	0.01
Oxycodone	17% (1082)	17% (644)	0.00
Oxycodone total dose (mg)	0 (0, 0) {3.8}	0 (0, 0) {3.8}	0.00
Female sex	41% (2635)	41% (1560)	0.00

Inferential statistical analyses were performed using Stata 17.0 (StataCorp, College Station, TX). The exact Clopper-Pearson method was used to calculate the two-sided 99% confidence interval for the percentage of patients where the dependent variable was one, versus zero. Standardized differences of predictors for the dependent variable were calculated using the stddiff command and reported using Cohen’s D (Table 1) [8]. The continuous variables’ standardized differences were calculated using their ranks.

Our starting hypothesis was that a few (<5%) of patients meeting inclusion criteria would have >90% probability for the dependent variable equaling one. In other words, fewer than 5% of intensive care unit patients who were alert and without delirium over two days (i.e., from 12 PM on day 1 through 12 PM on day three) would reliably (>90% probability) remain alert and without delirium over the next two days (i.e., a total of at least four days and five successive 12 PMs). Given that the hypothesis was the absence of a probability >90%, we did not segment the data into a training and testing dataset (i.e., our logistic regression models were deliberately overfit). In other words, our scientific goal was not the creation of a generalizable logistic regression model or decision tree for prediction, but, rather, to determine whether there was any potential for an interpretable statistical model to be useful for such predictions. If even an overfit model had unacceptable performance, there would be no point in further development. Thus, we report the estimated probabilities from the entire dataset. To evaluate if our finite population size was sufficient, we calculated the 99% confidence interval of the percentage of patients with >90% probability. The upper limit for the percentage of patients being less than 5% would show our hypothesis was satisfied. Importantly, we used 90% as the estimated probability of remaining in the intensive care unit for at least four days because many individual patients meeting this criterion would have substantially smaller lower prediction limits of the probability (e.g., probability >80%).

Our first sensitivity analysis used logistic regression with an estimated threshold probability of>80% rather than >90%. Because this was the logistic regression’s point estimate of 80%, many patients would have a significantly smaller lower prediction limit (i.e., the false positive rate for remaining in the intensive care unit >20% [9,10]). Thus, the intensive care unit clinicians would often see assessments made and treatments started for patients who would be discharged from the intensive care unit before potential benefits could either be assessed or realized. Therefore, our use of >80% estimated probability was to ensure that our conclusions were not strikingly dependent on the choice of a threshold of >90% estimated probability. Note that our use of 80% is not meant to suggest clinical or managerial relevance to applying a threshold probability >80%, because we doubt its relevance for that purpose [9,10].

Our second set of sensitivity analyses used several machine-learning classification methods, applying the >90% threshold probability of the patient remaining alert and without delirium in the intensive care unit, the same as for the primary analysis. The question asked was whether any such methods can achieve sufficiently greater sensitivity for the dependent variable equaling 1 without a large decrease in the positive predictive value (i.e., testing if the accuracy of classification was improved significantly, compared with logistic regression). To compare the methods meaningfully, we created a training dataset by selecting 70% of the cases at random and without replacement, fitting the models using hyperparameter tuning and cross-validation and, and then evaluating performance using the remaining 30% of cases. Comparisons were made pairwise by iteration with the best-performing (overfit) logistic regression model. The process was repeated 199 times to have a total of 200 random samples for each machine-learning method. The paired two-sided Student t-tests were Šidák corrected for the multiple comparisons to maintain the family-wise error rate at 0.01 for the single hypothesis that at least one method performed better than logistic regression. We used the mlr package and the command *makeLearner* from R version 4.2.1 (R Foundation for Statistical Computing, Vienna, Austria) under RStudio version 2022.07.02+576 (RStudio Team, Boston, MA). Learner classes included classif.logreg (logistic regression), classif.naiveBayes (naïve Bayes), classif.rpart (decision tree), classif.randomForrest (random forest), and classif.xgboost (extreme gradient boosting). Hyperparameters were tuned using the tuneParams command. Random splitting was performed using the sample_frac command from the dplyr package. The conf_mat command from the caret package was used to calculate the confusion matrices and the associated performance metric. Annotated computer code is available for readers at https://FDshort.com/Awake4Days.

## Results

Among the 10,314 patients who were alert and without delirium in an intensive care unit over the two-day period including three successive 12 PMs, 37% (3826) were alert, without delirium, and in the intensive care unit for at least five successive 12 PMs (99% CI 36% to 38%). Most of the other 6,488 patients had left the intensive care unit before the fifth 12 PM, with 92% (5,970) transferred to a hospital ward, and 2% (108) dying. Among the 10,314 patients, there was a median of 46 Riker scale scores (interquartile range 30 to 79, mean 67). There was a median of 7.0 per day (interquartile range 6 to 9, mean 7.4). Each patient had a median of 12 CAM-ICU and/or DOS scores (interquartile range 7 to 21, mean 17). There was a median of 2.0 scores per day (interquartile range 1 to 3, mean 2.0). Most assessments were made during one of the six daily multiples of four hours (i.e., 00:01 to 00:59, 04:01 to 04:59, 08:01 to 08:59, 12:01 to 12:59, 16:01 to 16:59, and 20:01 to 20:59), specifically 66% of the Riker scale scores (455,978/688,928) and 85% of the CAM-ICU and/or DOS scores (152,359/178,792).

For predicting the 37% of patients who remained alert, without delirium, and present in the intensive care unit for the next two days, there were six patient characteristics with Cohen’s D that were small [8] (i.e., >0.2 to 0.5) and 12 patient characteristics with Cohen’s D that were very small (i.e., >0.1 to 0.2) (Table 1). (None were moderate, >0.50 [8].) These patient characteristics were used in complete logistic regression, forward stepwise logistic regression, and backward stepwise logistic regression (Table 2). There were no more than two patients (0.02%) with an estimated probability >0.90 and 54 patients (0.5%) with an estimated probability >0.80 being alert, without delirium, and still in the intensive care unit two days later (Table 2). The same patient characteristics also were used in the decision tree classifier (Table 3). Even though these classification models were deliberately biased to result in artificially high performance, there were no patients (0%) with estimated probability >0.90 and no more than 60 patients (0.6%, 99% CI 0.4% to 0.8%) with estimated probability >0.80 (Table 3). Thus, at most 0.8% of the intensive care unit patients alert and without delirium for three successive 12 PMs (i.e., at least two days) had an estimated probability >0.80 of staying in the intensive care unit and being alert and without delirium for the next two successive 12 PMs (i.e., total at least four days). The 0.8% being less than 5.0% means that our hypothesis of lack of useful prediction from interpretable models (logistic regression, decision trees) was supported.

**Table 2:** Results of logistic regression models using the n=10,314 observations ^a^ The three continuous variables in Table 1 with Cohen’s D >0.20 (i.e., small) [8] had the same standardized difference when calculated using the corresponding binary variable because >86% of patients did not receive the medication. Therefore, although Table 1 has nine rows with Cohen’s D >0.20, we used the six binary variables in the logistic regression. Similarly, the one continuous variable in Table 1 with 0.20 > Cohen’s D >0.10 (i.e., very small) had the same standardized difference when calculated using the corresponding binary variable, because >91% of patients did not receive the medication. Therefore, although Table 1 has 16 rows with Cohen’s D >0.10, we used the 12 binary variables in the logistic regression. There were no variables with Cohen’s D >0.5 (i.e., moderate range) [8]. ^b^ The full model with 12 variables had the largest number of observed cases with estimated probabilities >0.90 and >0.80. The estimated area under the Receiver Operating Characteristic curve was 0.65.

% (count) estimated probability >0.90 (planned endpoint)	% (count) estimated probability >0.80 (sensitivity analysis)	Variables included those from Table 1 with Cohen’s D^a^	Stepwise logistic regression modeling	P-value criterion to enter variable into model	P-value criterion to remove variable from model	Logistic regression model
0.0% (1)	0.2% (21)	>0.20	Full	Not applicable	Not applicable	5 variables P <0.01, norepinephrine P=0.077
0.0% (0)	0.4% (38)	>0.20	Forward	P <0.05	P >0.10	5 variables
0.0% (1)	0.2% (21)	>0.20	Backward	P <0.05	P >0.10	5 variables
0.0% (0)	0.4% (38)	>0.20	Forward	P <0.01	P >0.05	5 variables
0.0% (0)	0.4% (38)	>0.20	Backward	P <0.01	P >0.05	5 variables
0.0% (2)	0.5% (54)	>0.10	Full	Not applicable	Not applicable	7 variables P <0.01^b^
0.0% (1)	0.5% (49)	>0.10	Forward	P <0.05	P >0.10	8 variables
0.0% (1)	0.5% (53)	>0.10	Backward	P <0.05	P >0.10	9 variables
0.0% (0)	0.3% (30)	>0.10	Forward	P <0.01	P >0.05	7 variables
0.0% (1)	0.5% (49)	>0.10	Backward	P <0.01	P >0.05	8 variables

**Table 3:** CART decision tree fit using regression Although CART nominally stands for Classification And Regression Trees, CART is the proper name of one such algorithm, that was used for this study. Our deliberate objective was to overfit the model if that resulted in more patients having predicted probabilities greater than 0.90. We were estimating the maximum potential percentage of patients with predicted probabilities exceeding 0.90. ^a^ The minimum of 51 cases is 0.5%, which was the tiny size, deliberately impractically small. However, the table shows that even that restriction resulted in no predicted probabilities even exceeding 0.80. ^b^ The three continuous variables in Table 1 with Cohen’s D >0.20 (i.e., small) [8] had the same standardized difference when calculated using the corresponding binary variable because >86% of patients did not receive the medication. Therefore, although Table 1 has nine rows with Cohen’s D >0.20, we used the six binary variables. Similarly, the one continuous variable in Table 1 with 0.20 > Cohen’s D >0.10 (i.e., very small) had the same standardized difference when calculated using the corresponding binary variable, because >91% of patients did not receive the medication. Therefore, although Table 1 has 16 rows with Cohen’s D >0.10, we used the twelve binary variables when creating the classification tree.

% (count) estimated probability >0.90, planned endpoint	% (count) estimated probability >0.80, sensitivity analysis	Minimum cases at end of tree branch^a^	Variables included those from Table 1 with Cohen’s D^b^	Count of variables included in decision tree
0% (0)	0.6% (60)	1	>0.20	6 of the 6 available
0% (0)	0% (0)	51	>0.20	1 of the 6 available, any hemodynamic infusion
0% (0)	0% (0)	1	>0.10	9 of the 12 available
0% (0)	0% (0)	51	>0.10	1 of the 12 available, any hemodynamic infusion

Table 4 shows results comparing four machine learning methods (each with six and 12 variables) and logistic regression (with 12 variables) versus logistic regression with the six variables having Cohen’s D >0.2. For all 10 combinations, patients were classified using the >0.90 probability threshold of staying in an intensive care unit alert and without delirium. The performance of the machine learning methods was not improved by including the six variables with Cohen’s D between 0.1 and 0.2. Random forest with the six patient characteristics had significantly greater accuracy than logistic regression for predicting patients staying in the intensive care unit, alert and without delirium, for four consecutive days conditional on being at least two days (adjusted P=0.0002, Table 4). However, the increase in accuracy among the 200 iterations was small, from 62.9% (standard deviation 0.7%) with logistic regression to 65.9% (0.8%) with random forest. The reason for the pairwise increase in accuracy being small (3.0%) was that although random forest had a much larger average sensitivity of 30.8% for predicting patients with probability >0.90, its positive predictive value for those patients averaged only 57.5%. Table 5 shows the relative performance for combinations of the six patient characteristics.

**Table 4:** Mean (standard deviation) of pairwise comparison among 200 iterations between nine machine learning methods and logistic regression using six patient characteristics For each of 200 iterations, the n=10,314 observations were selected at random and without replacement to be in the training dataset (70%) or test dataset (30%). All these 10,314 patients had remained in the intensive care unit alert and without delirium for at least two days. Logistic regression using six variables as in Table 2 was tuned using the training data and applied to the test data. Accuracy and sensitivity (recall) were calculated based on the predicted probability for each patient exceeding 90% to remain in the intensive care unit for at least two more days, alert and without delirium. For the same iteration, the difference was taken for each of the machine learning methods with the logistic regression result. The mean (standard deviation) of accuracy for logistic regression was 62.9% (0.7%), with sensitivity of 0.0% (0.0%). ^a^  The three continuous variables in Table 1 with Cohen’s D >0.20 (i.e., small) [8] had the same standardized difference when calculated using the corresponding binary variable, because >86% of patients did not receive the medication. Therefore, although Table 1 has nine rows with Cohen’s D >0.20, we used the six binary variables when performing the calculations for this Table 4. Similarly, the one continuous variable in Table 1 with 0.20 > Cohen’s D >0.10 (i.e., very small) had the same standardized difference when calculated using the corresponding binary variable, because >91% of patients did not receive the medication. Therefore, although Table 1 has 16 rows with Cohen’s D > 0.10, we used the 12 binary variables for our calculations.

Machine learning method	Numbers of variables^a^	Accuracy	Sensitivity (recall)
Naive Bayes	6	0.6% (0.2%)	3.0% (0.4%)
Decision tree	6	0.0% (0.0%)	0.0% (0.0%)
Random forest	6	3.0% (0.7%)	30.8% (3.1%)
XGBoost	6	0.0% (1.0%)	0.0% (0.1%)
Logistic regression	12	0.0% (1.1%)	0.2% (0.1%)
Naive Bayes	12	0.9% (1.1%)	5.0% (0.8%)
Decision tree	12	0.0% (1.1%)	0.0% (0.0%)
Random forest	12	2.2% (1.2%)	22.1% (4.9%)
XGBoost	12	0.0% (1.1%)	0.2% (0.3%)

 

**Table 5:** Example from the first of 200 iterations comparing random forest and logistic regression each with six patient characteristics used in the model With six binary patient characteristics, there are 64 possible combinations. Among the 3,094 patients in the test dataset, there were 21 of these 64 combinations with at least one patient. The eight combinations shown in this table are the eight combinations each with at least 17 patients among the 3,094 patients in the test dataset. These eight combinations accounted for 98% of all patients, where 98% was the sum of the percentages from the second row. Using these eight combinations shown, logistic regression would have accuracy of 63%, where 63% = (1,925 patients from sum of 14th row)/(3,041 patients from sum of first row). Random forest would have accuracy of 66% = (2,012 patients from sum of last row)/(3,041 patients from sum of first row). The slightly greater accuracy of random forest shows that this example was realistic, even though being from just one of the 200 iterations and showing only eight of the 64 possible combinations.

Combination	1	2	3	4	5	6	7	8
Patients in test dataset with combination of factors	2154	278	260	199	55	45	33	17
% of the 3094 patients in the test dataset	70	9	8	6	2	1	1	1
Combination with: Any hemodynamic infusion	0	0	0	1	1	1	1	1
Combination with: Any ventilatory support	0	1	1	0	1	0	0	1
Combination with: Optiflow nasal high flow therapy	0	1	0	0	0	0	0	1
Combination with: Norepinephrine infusion	0	0	0	1	1	0	0	1
Combination with: Milrinone infusion	0	0	0	0	0	1	0	0
Combination with: Epinephrine infusion	0	0	0	0	0	0	1	0
Patients present for four days, alert no delirium	636	153	123	114	32	29	20	9
Present for four days among those in column (%)	30	55	47	57	58	64	61	53
Logistic regression estimated probability	30	54	46	51	67	64	60	74
Logistic regression probability with all data (Table 2)	30	54	45	51	67	65	59	74
Logistic regression patients with probability >0.90	0	0	0	0	0	0	0	0
Logistic regression patients accurately predicted	1518	125	137	85	23	16	13	8
Random forest patients with probability >0.90	0	278	0	199	135	54	45	32
Random forest sensitivity (%)	0	100	0	100	100	100	100	100
Random forest positive predictive value (%)		55		57	58	64	61	53
Random forest patients accurately predicted	1518	153	137	114	32	29	20	9

## Discussion

Slightly more than one-third of intensive care unit patients who were alert and without delirium for two consecutive days remained in the intensive care unit alert and without delirium for the next two days. Our key result was that despite using 45 variables and six statistical methods, no combination came close to predicting ≥5% of the patients each with a substantial (>90%) probability of staying in the intensive care unit for the next two days. Many patients were discharged from the intensive care unit before 96 hours had elapsed. One implication is that assessment of dignity-related distress and intervention (e.g., changing clinicians’ interaction with and support of the patient) should be taken from the perspective of the entire hospitalization, rather than as an isolated activity in the intensive care unit. While the intensive care unit team may assess distress and start individualized intervention, delivery of the intervention probably would best be delivered by or coordinated with a different specialty team that would assure continuity of care at least through hospital discharge (e.g., palliative care, spiritual care, psychological nursing, or occupational therapy).

We used three successive 12 PMs (i.e., first two consecutive days) as inclusion criteria to predict the next two successive 12 PMs (i.e., second two consecutive days). This choice of our studying five successive 12 PMs assured the clarity and generalizability of our results. We initially selected 12 PM because this particular time often is used in hospital management studies to denote bed availability [11]. A strength of our study is that our conclusion is especially dependable because there not only is variability in patient condition as we studied but also variability in when the specialty consultative team would be available. An earlier study of palliative care consults found the time course for patients to be seen to be in units of days, not one or two hours earlier or later than 12 PM [12]. Furthermore, an assessment for psychological distress would often be a lower priority than the treatment of patients with confirmed symptoms or the assessment of patients who will be discharged that day [11]. Therefore, often our studied patients would be seen later in the workday, after 12 noon. In a study of preoperative consultations among hospitalized patients, assessments were completed by 6 PM [13]. Thus, the expected large variability among organizations in the workflow of consultations further supports our conclusions.

Our study is limited to being from a single hospital. However, three observations provide evidence for confidence in the validity and generalizability of our specific results. First, probably because our inclusion criteria included that patients had to be alert and without delirium for at least 48 consecutive hours, too few (6%) of the patients developed delirium over the next 48 hours for the clinical or pharmaceutical risk factors of delirium to have affected our results. In other words, in retrospect, our results were functionally a study of predicting patient discharges, not a study of new-onset delirium. The times of patient discharge from intensive care units are known retrospectively without error for nearly every patient. Second, at a different hospital, Levin and colleagues previously used >75 variables in a random forest model to predict for a telemetry unit’s daily early morning round whether the patient would be discharged by the end of the next day [13]. Their accuracy in predicting patient discharge was 64% [14], matching the low accuracy of our models. Second, the mean 63% accuracy of logistic regression among our 200 iterations of resampling reflected nothing more than a sensitivity of 0% for patients having a 90% probability or greater of remaining in the intensive care unit for at least four days while alert and without delirium (Tables 4, 5). In other words, the accuracy equaled one minus the 37% prevalence of patients who were alert and without delirium for two days and remained so in the intensive care unit for another two days. That prevalence not depending on modeling or statistical assumptions shows that our results are unlikely biased estimates. Finally, third, our inclusion criteria were such that the binary outcome of one patient had essentially no chance of affecting the outcome of another patient (i.e., statistical independence as assumed likely was satisfied).

## Conclusions

Although most intensive care unit patients who are alert and without delirium for two days are discharged from the unit before two more days have elapsed, many remain in the intensive care unit alert and without delirium for at least two days. Neither logistic regression nor any of the four machine learning methods evaluated came close to predicting accurately which patients would remain in the intensive care unit. Patients’ dignity-related distress and intervention should be considered from the perspective of the entire hospitalization, not as an intensive care unit activity limited to intensive care unit physicians and nurses.
